# Complete Genome Sequence of a *Mucilaginibacter* sp. Strain Isolated from Estuarine Soil Contaminated with Mine Tailings from the Samarco Disaster at Fundão Dam

**DOI:** 10.1128/MRA.00779-21

**Published:** 2021-10-14

**Authors:** Ana L. S. Vasconcelos, Thaiane Defalco, Armando C. F. Dias, Leticia Barrientos, Angelo F. Bernardino, Fernando Dini Andreote, Kattia Núñez-Montero

**Affiliations:** a Departmento de Solos e Nutrição de Plantas, Escola Superior de Agricultura “Luiz de Queiroz,” Universidade de São Paulo, São Paulo, Brazil; b Laboratorio de Biologia Molecular Aplicada, Centro de Excelência em Medicina Traslacional, Universidad de La Frontera, Temuco, Chile; c Departamento de Oceanografia, Universidade Federal do Espírito Santo, Vitória, Brazil; d Centro de Investigación en Biotecnologia, Instituto Tecnológico de Costa Rica, Cartago, Costa Rica; Queens College CUNY

## Abstract

We report the complete genome sequence of *Mucilaginibacter* strain 21P, which was isolated from estuarine soil contaminated with mine tailings from the Samarco disaster, which occurred in 2015 in Brazil. The genome sequence comprised 4,739,655 bp, with a G+C content of 43.2%, and harbors multiple antibiotic and metal resistance genes.

## ANNOUNCEMENT

The genus *Mucilaginibacter* belongs to the family *Sphingobacteriaceae*, which are nonmotile, rod-shaped, and exopolysaccharide-producing (EPS) bacteria (1, 2). Members of this genus are widespread in the environment (3–5) and usually contain resistance to potential toxic metal(loid)s and antimicrobials (6–8).

Here, we report the complete genome sequence of *Mucilaginibacter* strain 21P, isolated from an estuarine soil contaminated with iron mine tailings from the Samarco disaster, which occurred in 2015 in Minas Gerais State, Brazil. The soil core was collected in December 2018 (24 months after the disaster) from the Rio Doce Estuary, Brazil (19°23′28″S, 40°04′20″W). For isolation, 5 g of the core soil (5 to 10 cm depth) was homogenized; serial dilutions were plated onto 10% tryptic soy (TS) agar (Merck) supplemented with nystatin (1 mg/ml) and Mn (1.6 mg/ml) and incubated at 30°C for 24 h, as previously described (9). The separated colonies were isolated onto new plates, and pure isolates were cultivated in TS broth (Merck) with agitation at 150 rpm for 24 h for DNA extraction using the Wizard genomic DNA purification system (Promega, The Netherlands). Genomic DNA was sequenced using the MiSeq (Illumina, Inc., San Diego, CA, USA) and MinION (Oxford Nanopore Technologies [ONT], UK) platforms. The Illumina library was prepared using the Nextera XT DNA library prep kit (Illumina, Inc.) and sequenced using the paired-end method, with an average read size of 350 bp in a 2 × 150-bp sequencing run, for a total of 500,636 Illumina reads. The MinION library was prepared using the rapid barcoding kit (SQK-RBK004; Oxford Nanopore Technologies); the sequencing was performed using MinKNOW software, followed by base calling and conversion of the raw data to FASTQ format using Guppy v.3.6.0 (https://staff.aist.go.jp/yutaka.ueno/guppy/). The recovered data resulted in 39,386 ONT reads with a mean read length of 4,217 bp and an *N*_50_ value of 7,217 bp.

Low-quality ONT reads and adapters (Phred score, <10; length, <5,000) were trimmed using Porechop v.0.2.4 and NanoFilt v.2.8.0, respectively (10). The Illumina reads were filtered using Fastp v.0.20.1 (11) with default parameters and checked using FastQC (7). Hybrid *de novo* genome assembly was performed using Unicycler v.0.4.8 software (12), which includes removal of overlapping sequences using the SPAdes optimizer (13), genome polishing using Pilon, a final circularization by connecting its end to its start, and a rotation where the *dnaA* or *repA* alleles were found using TBLASTN (12). The annotation was performed using the Prokka v.1.14.6 tool (14). Default parameters were used for all software unless otherwise specified.

The genome comprised 4,739,655 bp, with a G+C content of 43.2% in a unique contig with 4,334 coding sequences, including 53 rRNA genes and antibiotic/metal resistance genes, including *arsCM*, *zraR*, *mnmACEG*, *corA*, *gyrA*, *blc*, *ampGH*, and *folPA*. The average nucleotide identity (ANI) was calculated against the available genome sequences from *Mucilaginibacter* (32) using the JSpeciesWS online server (15). The results were below the species threshold (>95%), with a maximum of 74.1% for Mucilaginibacter rigui (6), suggesting that this isolate might be a new species.

### Data availability.

The complete genome sequence of *Mucilaginibacter* sp. strain 21P can be found in NCBI GenBank under the accession number GCF_019331605.1. The raw Illumina and MinION reads are available under the SRA accession numbers SRX11522297 and SRX11522298, respectively.
